# 123 Cross-Cutting Prevention Tool for Early Identification of Youth Firesetting

**DOI:** 10.1093/jbcr/iraf019.123

**Published:** 2025-04-01

**Authors:** Karla Klas, Ann Yeomans, Anthony Campagna

**Affiliations:** University of Michigan, Michigan Medicine Trauma Burn Center; CT. Dept. of Children and Families; Greater New Haven Counseling and Family Therapy Center

## Abstract

**Introduction:**

Youth firesetting (YFS) is a significant community risk that is often overlooked and underreported. Despite its negative impact, landscape analysis shows sporadic early identification with inconsistent interventions for at-risk youth. This project aimed to address the gap by creating a novel comprehensive tool to identify firesetting youth earlier and more consistently.

**Methods:**

An international multidisciplinary group of subject matter experts convened to develop a brief YFS screening tool, subsequently named the Firesetting & Fire Safety Identification Tool (FFIT). Key risk factors, cross-cutting protective factors and related critical “triage” questions were identified via a literature search and included in FFIT. A flowchart, data dictionary and structured decision-making guide were created to prompt appropriate follow-up and intervention pathways based on the tool’s 10 questions. FFIT underwent a 2-year iterative process of piloting testing with: Phase 1 - key stakeholders; Phase II - vetted YFS practitioners; and Phase III - large network of international risk reduction professionals who tested the tool with 3 specific YFS case studies.

**Results:**

An initial landscape analysis survey (n=346) found no existing early identification tool and a general lack of knowledge on evidence-based YFS interventions among community adults. All Phase I respondents (n=9) reported FFIT was directly helpful and vastly needed in their prevention work. Phase II YFS practitioners (n=37) revealed variability in question interpretation and confusion in overall flow. Hence to enhance sensitivity and specificity, the data dictionary, tool questions and guide were modified. To improve ease and applicability of use, a structured decision-making flowchart was created to be customizable with local resources and country-specific terminology. Phase III results (n=96) involved 46 respondents from 46 unique organizations with 50% from the fire service. Of the complete responses (n=82), >90% showed appropriate use and application of FFIT with even distribution across the 3 case studies. Qualitative evaluation results reveal FFIT establishes clear standardized YFS terminology, takes the “gut feel” out of screening, is greatly needed and can be completed by any adult without specialized training.

**Conclusions:**

FFIT is a novel and valuable tool provided at no cost for early identification and intervention of youth at risk for firesetting behaviors. Pilot testing demonstrated its feasibility, reliability and relevance across various settings. By providing a structured approach, FFIT empowers caregivers and professionals to identify and address YFS early, thereby preventing future fires and improving safety outcomes for at-risk youth and communities.

**Applicability of Research to Practice:**

FFIT can be widely incorporated into communities to further investigate YFS prevalence, correlates and the effectiveness of prevention programs.

**Funding for the Study:**

N/A

